# Multiple pleural nodules diagnosed as IgG4-related disease: a case report

**DOI:** 10.1186/s40792-021-01166-y

**Published:** 2021-04-07

**Authors:** Yoshihito Iijima, Shun Iwai, Nozomu Motono, Katsuo Usuda, Akihiro Shioya, Shingo Takeuchi, Shigeki Yamagishi, Kiyoshi Koizumi, Sohsuke Yamada, Hidetaka Uramoto

**Affiliations:** 1 Department of Thoracic Surgery, Kanazwa Medical University, 1-1 Daigaku, Uchinada-machi, Kahoku-gun, 920-0293 Ishikawa, Japan; 2 Department of Pathology and Laboratory Medicine, Kanazwa Medical University, Ishikawa, Japan; 3 Department of Thoracic Surgery, Tomei Atsugi Hospital, Kanagawa, Japan; 4 Department of Thoracic Surgery, Aidu Chuo Hospital, Fukushima, Japan

**Keywords:** IgG4-related disease, Multiple pleural nodules, Thoracoscopy

## Abstract

**Background:**

Immunoglobulin G4 (IgG4)-related diseases are characterized by abnormal IgG4 levels, swelling, and marked infiltration and fibrosis of the lymphocytes and IgG4-positive plasma cells, causing hypertrophic lesions or nodules. The cause is currently not well understood. IgG4-related diseases involving lesions limited to the pleura are extremely rare. Herein, we report an IgG4-related disease presenting with multiple pleural nodules confirmed by thoracoscopic surgical biopsy.

**Case presentation:**

A 74 year-old man was referred to our department for definitive diagnosis of multiple pleural nodules after 1 year of follow-up. Computed tomography of the chest revealed multiple pleural nodules, while 2-deoxy-2-( ^18^F)-fluorodeoxyglucose positron emission tomography imaging exhibited tracer accumulation in the nodules. A thoracoscopic surgical biopsy was performed. Histopathological examination revealed hyalinized fibrous tissue with a high degree of plasma cell-based inflammatory cell infiltration. Immunohistochemically, IgG4-positive cells were conspicuous, accounting for 70.5% of the plasma cells. The postoperative serum IgG4 concentration was 289 mg/dL. We diagnosed the patient with an IgG4-related disease with multiple pleural nodules. The postoperative course was good, and the patient is currently being followed up.

**Conclusion:**

IgG4-related disease should be considered in cases presenting with multiple pleural nodules.

## Background

Immunoglobulin G4-related disease (IgG4-RD) is attracting increasing attention as a relatively new disease originating in Japan [1, 2]. In addition to immune abnormalities and high circulating levels of serum IgG4, its manifestations include a swelling of various organs and the synchronous/metachronous appearance of nodules/hypertrophic lesions caused by marked infiltration and fibrosis of lymphocytes and IgG4-positive plasma cells. The cause is currently unknown, and IgG4-RD with lesions confined to the pleura is extremely rare. We report a case of an IgG4-RD presenting with multiple pleural nodules confirmed by thoracoscopic surgical biopsy.

## Case presentation

**Fig. 1:**
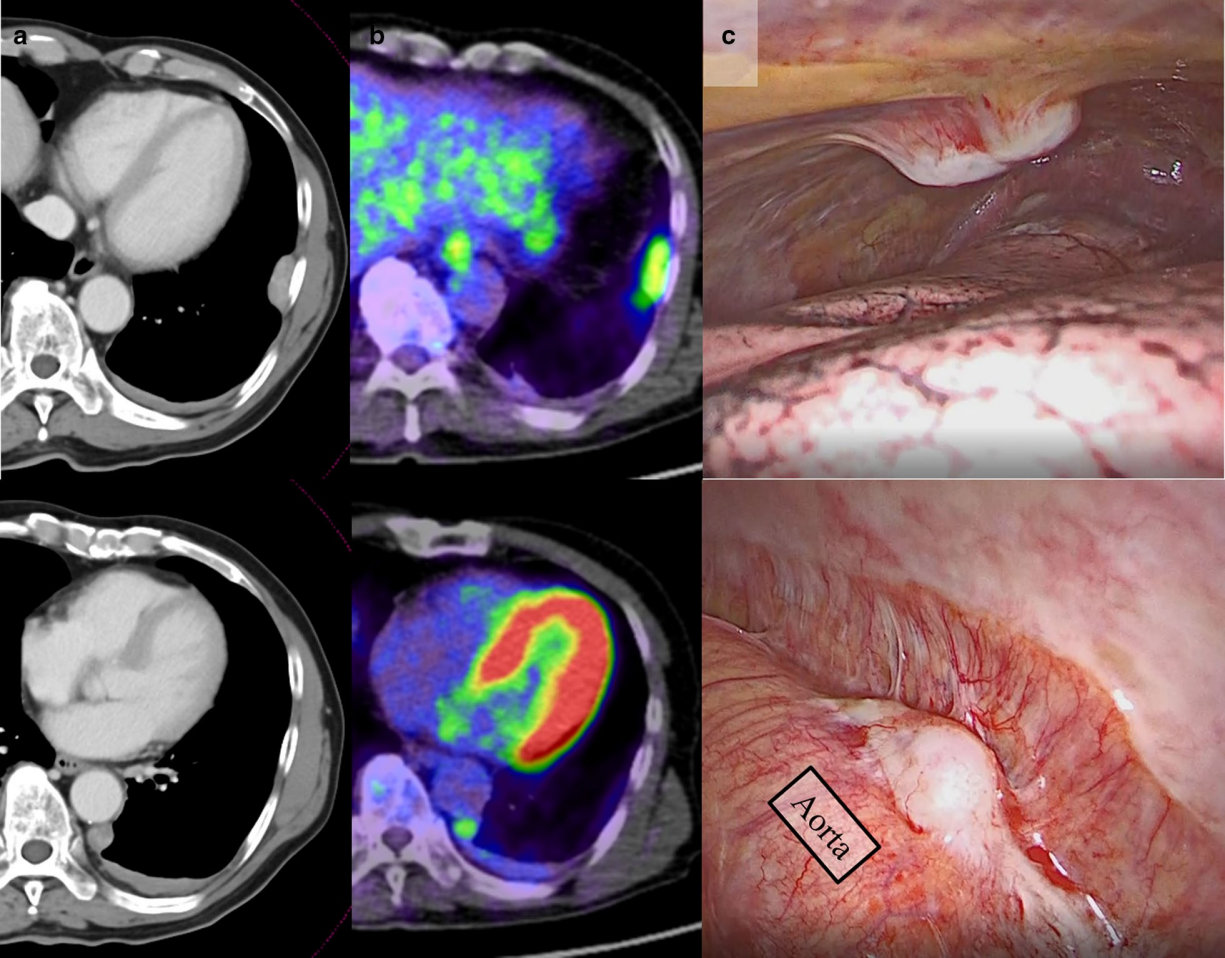
Computed tomography (CT) and ^18^F-fluorodeoxyglucose (FDG) positron emission tomography (PET)/CT. **a** CT of the chest revealed multiple pleural nodules, including a 2.7 × 1.8 × 1.1 cm nodule inside the mid-axillary line of the 8th rib and a 1.3 × 1.2 × 2.9 cm nodule at the height of the 9th thoracic vertebral body on the dorsal side of the descending aorta. **b** FDG-PET/CT maximum standardized uptake values of the lateral nodule and the nodule of the dorsal side of descending aorta were 4.93 and 3.18, respectively. **c** Intraoperative images of the nodule inside the mid-axillary line of the 8th rib and the nodule at the height of the 9th thoracic vertebral body on the dorsal side of the descending aorta

**Fig. 2:**
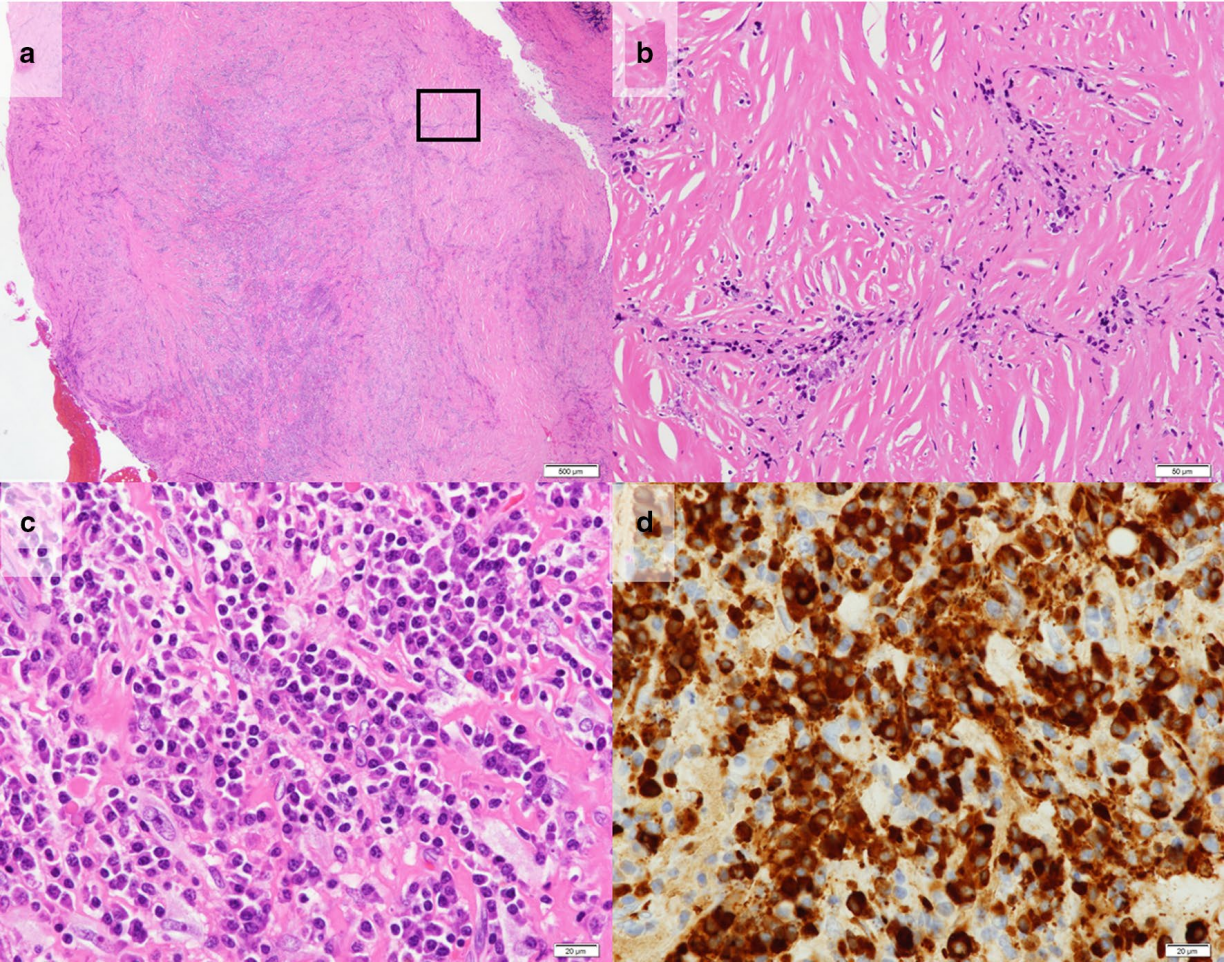
Histopathological and immunohistochemical findings. The nodule consisting of hyalinized fibrous tissue, with a high degree of plasma cell-based inflammatory cell infiltration. A storiform pattern was observed in some parts of the fibrosis. Obstructive phlebitis was not apparent. **a** Hematoxylin and eosin (H&E) staining, × 2 magnification. The black rectangle in (**a**) is the same area as shown in (**b**). **b** H&E staining, × 20 magnification. **c** H&E staining, × 40 magnification. **d** IgG4- positive plasma cells were conspicuous, accounting for 70.5% of cells

A 74 year-old asymptomatic man with no history of smoking was referred to our department with a definitive diagnosis of multiple pleural nodules. The patient had a career working as a welder in the iron works. He had a history of hypertension, hyperuricemia, and chronic kidney disease. Chest X-ray revealed an abnormal shadow in the lower left lung field, and the patient was under follow-up with the respiratory medicine department for 1 year. Laboratory test results for tumor markers, including the carcinoembryonic antigen, squamous cell carcinoma antigen, cytokeratin-19 fragment, sialyl-Lewis X antigen, and pro-gastrin-releasing peptide, were within the normal ranges. Computed tomography (CT) of the chest (Fig. 1a) revealed multiple pleural nodules, including a 2.7 × 1.8 × 1.1 cm nodule inside the mid-axillary line of the 8th rib and a 1.3 × 1.2 × 2.9 cm nodule at the height of the 9th thoracic vertebral body on the dorsal side of the descending aorta. These nodules exhibited a heterogeneous pale contrast. During the course of 1 year, the nodules showed a slight tendency towards increased prominence, and a small amount of pleural effusion developed over time. Neither lung lesion nor hilar/mediastinal lymphadenopathy was observed. A whole-body positron emission tomography (PET)/CT scan using 2-deoxy-2-( ^18^F)-fluorodeoxyglucose demonstrated maximum standardized uptake values of 4.93 and 3.18 in the lateral nodule and the nodule at the dorsal side of the descending aorta, respectively (Fig. 1b). No clear tracer accumulation outside the pleural lesions was observed. A malignant tumor could not be ruled out, and a surgical biopsy was planned. Thoracoscopy revealed multiple gray-white pleural nodules (Fig. 1c). A part of the 8th intercostal nodule was resected. The submitted tissue specimen showed a variably thickened and hyalinized fibrous plaque with focal inflammatory granulation and no apparent neoplastic components. The intraoperative frozen section presented with no evidence of malignancy. We judged that the other nodules had the same characteristics, and the operation was terminated. Histopathologically, the resected nodule was found to consist of hyalinized fibrous tissue with a high degree of plasma cell-based inflammatory cell infiltration (Fig. 2a–c). A storiform pattern was observed in a region of the fibrotic parts. Obstructive phlebitis was not apparent. Immunohistochemically, IgG4-positive plasma cells were conspicuous (Fig. 2d). The IgG4/CD38 positive plasma cell ratio was evaluated in the same five fields at a high magnification, with IgG4-positive plasma cells accounting for 70.5% of the cells (1,555/2,206 in total). A scattered distribution of spindle-shaped cells weakly positive for calretinin, WT1, D2-40, CAM5.2, AE1/AE3, and CK5/6 was observed. However, there were no positive findings suggestive of neoplastic changes, and no indication of mesothelioma. Evaluation for the BER-EP4, MOC-31, and p40 biomarkers yielded negative results, while that for beta-catenin yielded positive results in the cytoplasm; desmoids were not observed. The postoperative serum IgG4 level was 289 mg/dL, the rheumatoid factor level was not elevated, and the samples were negative for antinuclear antibodies. Based on these observations, we diagnosed him with IgG4-RD with multiple pleural nodules. The postoperative course was good and the patient is currently being followed up.

## Discussion

IgG4-RD is a newly recognized fibroinflammatory condition characterized by tumefactive lesions, a dense lymphoplasmacytic infiltrate rich in IgG4-positive plasma cells, storiform fibrosis, and elevated serum IgG4 concentrations [3]. Based on the reports of high serum IgG4 levels in autoimmune pancreatitis from Japanese research groups, lesions that infiltrate systemic organs were also found to exist in other diseases, such as the Miklicz disease [1, 2]. Recently, IgG4-RD has been recognized as a common systemic disorder. In 2011, comprehensive diagnostic criteria for IgG4-RD were proposed by the Study Group of Intractable Disease from the Ministry of Health, Labour and Welfare of Japan [4]. Furthermore, the diagnostic criteria for IgG4-related respiratory disease (IgG4-RRD), which is a form of IgG4-RD involving respiratory lesions, were published in the journal of the Japanese Society of Respiratory Society in 2015 [5, 6].

The case was diagnosed with IgG4-RD with multiple pleural nodules based on the histological features and the postoperative high serum IgG4 levels, according to the diagnostic criteria for IgG4-RRD. The patient had a career working as a welder in the iron works, with an unknown history of asbestos exposure. There are reports of pleuritis related to IgG4-RRD in asbestos-exposed individuals [7], and clinically, the presence of IgG4-RRD should help exclude the diagnosis of malignant mesothelioma. In addition, there are 13 reports, including the present case, on IgG4-related pleural disease without extrathoracic lesions (Table 1). All the reported patients were male, with an average age of 65.6 years (range: 16–81 years). The mean reported serum IgG4 level was 414.0 mg/dL (range: 142–1650 mg/dL). Pleural effusion was observed in 11 cases, and only five cases had pleural lesions without pulmonary lesions. Nine patients were treated with steroids, while four cases were managed by observation.

**Table 1:** IgG4-related pleural disease without extrathoracic lesions

No	Age (years)	Sex	Serum IgG4 (mg/dL)	Pleural effusion	Pleural thickening	Pleural nodules	Lymphadenopathy	Other thoracic findings	Therapy	References
1	78	M	483	Both	Mild	Dense granular	–	–	Observation	[13]
2	69	M	277	Both	+	–	+	Bronchiectasis with bronchial wall thickening, centrilobular nodules in the bilateral lungs	PSL	[14]
3	74	M	201	Right	+	-	–	Patchy lesions of the right lung, multiple nodules	PSL	[15]
4	66	M	148	–	–	+	–	Consolidative lesion at the right lower lobe	Observation	[16]
5	16	M	1650	Both	NA	NA	–	Bronchial wall thickening, soft tissue density at the anterior mediastinum	PSL	[17]
6	70	M	224	Right	–	–	+	Mediastinal adenopathies	PSL	[18]
7	64	M	280	Right	+	–	–	–	Oral steroid	[19]
8	70	M	1030	Right	Diffuse	–	–	–	PSL	[20]
9	65	M	164	Both	+	–	–	Left pneumothorax, bronchiectasis, atelectasis	PSL	[21]
10	81	M	233	Both	–	–	Mild	Subpleural GGO, consolidation of the left lobe	PSL	[22]
11	46	M	142	Both	–	+	−	–	PSL	[23]
12	80	M	261	Left	Diffuse	-	−	Right enveloping effusion, calcification of the right pleura	Observation	[24]
13	74	M	289	–	+	+	−	–	Observation	This case

*M* male, *GGO* ground glass opacity, *PSL* prednisolone, *NA* not available

In IgG4-RRD, the goal of the treatment is to maintain the lung function. Though there is no current consensus on the indication of treatment for IgG4-RRD, systemic corticosteroid therapy is recommended for patients with symptoms and with a high disease activity [8, 9]. The treatment of IgG4-RRD complies with the Japanese consensus guidelines for the treatment of autoimmune pancreatitis. Initial treatment includes 0.6 mg/kg/day of prednisolone. After continuous administration for 2–4 weeks, the dose is gradually reduced by 5 mg every 1–2 weeks for 2–3 months. The recommended maintenance dose is 2.5–5 mg/day [10]. In the present case, only pleural lesions were found, with no lesions detected in other organs and no respiratory symptoms observed. Therefore, the present follow-up policy was adopted. A rise in the IgG4 levels would suggest IgG4-RD involving other organs. Specifically, cases involving pleural lesions are associated with reports of pericardial lesions [11, 12], and thus, caution is warranted.

The patient was diagnosed with IgG4-RD with multiple pleural nodules. He is being followed up closely following the biopsy, and a steroid treatment has been planned if the IgG4 levels rise or pleural effusion occurs.

## Conclusion

IgG4-RD should be considered in cases presenting with multiple pleural nodules.

## Abbreviations

CT: Computed tomography
IgG4-RD: IgG4-related diseases
IgG4-RRD: IgG4-related respiratory disease
PET: Positron emission tomography
